# P-310. Evolutionary dynamics of the Chilean-Cordobes MRSA clone allowed the emergence of new MRSA clones in Chile

**DOI:** 10.1093/ofid/ofae631.513

**Published:** 2025-01-29

**Authors:** Jose R W Martínez, Amy Campbell, Maria Spencer, Lina M Rivas, Lorena Diaz, Ahmed Moustafa, Araos Rafael, Patricia Garcia, Cesar A Arias, Paul J Planet, José M Munita

**Affiliations:** Genomics & Resistant Microbes (GeRM), Instituto de Ciencias e Innovación en Medicina, Facultad de Medicina Clínica Alemana, Universidad del Desarrollo, Chile; Millennium Initiative for Collaborative Research on Bacterial Resistance (MICROB-R), Santiago, Region Metropolitana, Chile; The Children’s Hospital of Philadelphia & University of Pennsylvania, Philadelphia, Philadelphia, Pennsylvania; Genomics & Resistant Microbes (GeRM), Instituto de Ciencias e Innovación en Medicina, Facultad de Medicina Clínica Alemana, Universidad del Desarrollo, Chile; Millennium Initiative for Collaborative Research on Bacterial Resistance (MICROB-R), Santiago, Region Metropolitana, Chile; Genomics & Resistant Microbes (GeRM), Instituto de Ciencias e Innovación en Medicina, Facultad de Medicina Clínica Alemana, Universidad del Desarrollo, Chile; Millennium Initiative for Collaborative Research on Bacterial Resistance (MICROB-R), Santiago, Region Metropolitana, Chile; Univerdidad del Desarrollo, Santiago, Region Metropolitana, Chile; The Children’s Hospital of Philadelphia & University of Pennsylvania, Philadelphia, Philadelphia, Pennsylvania; Universidad del Desarrollo, Santiago, Region Metropolitana, Chile; Pontificia Universidad Catolica de Chile, Santiago, Region Metropolitana, Chile; Houston Methodist and Weill Cornell Medical College, Houston, TX; The Children’s Hospital of Philadelphia & University of Pennsylvania, Philadelphia, Philadelphia, Pennsylvania; Clínica Alemana - Universidad del Desarrollo, Santiago, Chile

## Abstract

**Background:**

The global spread of methicillin-resistant *Staphylococcus aureus* (MRSA) is associated with distinct clones predominating in specific geographical regions. Available evidence suggests that the Chilean-Cordobes clone (ChC), an ST5-SCC*mec*I lineage, has predominated in Chilean hospitals ever since its first description in 1998. However, we recently reported a gradual decrease in this lineage over time, with frequencies dropping from 94% (2000) to 52% (2016). To understand this clonal replacement event, we aimed to reconstruct the genomic evolution of the ChC in Latin America.

Characterization of clades and sub-lineages of the ChC clone.

**Figure 1.**
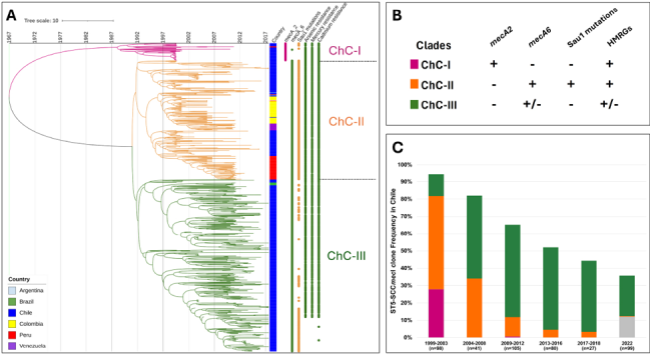
Characterization of clades and sub-lineages of the ChC clone. A) Maximum clade credibility molecular clock analysis of the 596 MRSA genomes. A Bayesian molecular clock analysis was performed with BEAST using the isolation date of each genome as calibrator. The vertical lines show the scale of years. The colored strip showed the country of origin. The dots show the presence of mecA genotypes, heavy metal resistance genes (HMRGs), and SNPs in Sau1. The branches are colored based on the sub-lineage identification: ChC-I (magenta), ChC-II (orange), and ChC-III (green). B) Characterization of the ChC clades. The presence of each genetic element considered to characterize the clades are marked by a + symbol. C) Relative frequency of the ChC sub-lineages in Chile over time. The colored bars represent the relative frequency of each sub-lineage: ChC-I (magenta), ChC-II (orange), ChC-III (green), and unclassified (gray).

**Methods:**

A total of 596 ChC MRSA isolates obtained from 6 Latin American countries between 1999-2018 were whole-genome sequenced and characterized using a Bayesian molecular clock analysis (MCA). Sub-lineages (SLs) observed in major divergence events were further characterized. Additionally, the frequency of each SL observed in our 1999-2018 collection was assessed in an independent group of genomes from 274 Chilean clinical MRSA isolates collected in 2022.

**Results:**

Our MCA of ChC MRSA revealed 3 clades strongly related with different genomic traits, including: i) mecA genotype, ii) heavy metal resistance genes (HMRGs), and iii) changes in the Sau1 restriction-modification system (Sau1) (Fig. 1A). Each clade was classified as sub-lineage: ChC-I, ChC-II, and ChC-III (Fig. 1B). All the genomes from Argentina, Colombia, and Venezuela – countries where ChC was largely replaced - belonged to the ChC-II SL and harbored a Sau1 mutation (Fig. 1A). On the other hand, genomes obtained from Chile distributed in different SLs and exhibited a dynamic interchange over time (Fig. 1C). While between 1999-2003 most of the genomes belonged to SLs ChC-I and ChC-II, both SLs virtually disappeared in 2013-2018, period were >90% ChC genomes corresponded to ChC-III (Fig. 1C). In 2022, a majority of ChC MRSA had genomic features compatible with the ChC-III SL (Fig. 1C). Finally, the MCA revealed an evolutionary event within ChC-III dated in 2005, which was accompanied by the loss of HMRGs (Fig. 1A)

**Conclusion:**

We describe the time evolution of several ChC clades, giving rise to sub-lineages that seem to play a role in the loss of dominance of the ChC MRSA clone in Chile and a consequent change in its epidemiology in the country.

**Disclosures:**

**José M Munita, MD**, MSD: Grant/Research Support|Pfizer: Grant/Research Support

